# Effects of Sampling Frequency on Human Activity Recognition with Machine Learning Aiming at Clinical Applications

**DOI:** 10.3390/s25123780

**Published:** 2025-06-17

**Authors:** Takahiro Yamane, Moeka Kimura, Mizuki Morita

**Affiliations:** 1 Department of Biomedical Informatics, Graduate School of Interdisciplinary Science and Engineering in Health Systems, Okayama University, Okayama 700-8530, Japan; pwf454co@s.okayama-u.ac.jp; 2 Faculty of Health Sciences, Okayama University Medical School, Okayama 700-8558, Japan; p7zt0ndt@s.okayama-u.ac.jp

**Keywords:** wearable devices, machine learning, human activity recognition, sampling frequency, digital health, digital biomarkers

## Abstract

Human activity recognition using wearable accelerometer data can be a useful digital biomarker for severity assessment and the diagnosis of diseases, where the relationship between onset and patient activity is crucial. For long-term monitoring in clinical settings, the volume of data collected over time should be minimized to reduce power consumption, computational load, and communication volume. This study aimed to determine the lowest sampling frequency that maintains recognition accuracy for each activity. Thirty healthy participants wore nine-axis accelerometer sensors at five body locations and performed nine activities. Machine-learning-based activity recognition was conducted using data sampled at 100, 50, 25, 20, 10, and 1 Hz. Data from the non-dominant wrist and chest, which have previously shown high recognition accuracy, were used. Reducing the sampling frequency to 10 Hz did not significantly affect the recognition accuracy for either location. However, lowering the frequency to 1 Hz decreases the accuracy of many activities, particularly brushing teeth. Using data with a 10 Hz sampling frequency can maintain recognition accuracy while decreasing data volume, enabling long-term patient monitoring and device miniaturization for clinical applications.

## 1. Introduction

The rapid advancement of wearable technology has enabled the continuous acquisition of biometric data outside of clinical settings. In particular, human activity recognition (HAR) using wearable accelerometers is a promising tool in clinical contexts, where understanding the relationship between physical activity and symptom onset is crucial for diseases such as chronic obstructive pulmonary disease (COPD) and arrhythmia. COPD is a lung disorder in which patients experience shortness of breath, even during daily light activities [1]. The occurrence of shortness of breath and the corresponding activity types are vital to assess COPD severity. Arrhythmia is a cardiac disorder characterized by palpitations, shortness of breath, and dizziness. The emergence of these symptoms and associated activity types are important to diagnose arrhythmia using Holter electrocardiography (ECG) [2]. However, current diagnostic methods for these conditions, such as the 6 min walk test and Holter ECG, pose challenges due to their burden on patients and lack of objectivity [3,4,5]. Therefore, these clinical applications require accurate yet unobtrusive monitoring systems that minimize patient burden while providing objective and real-time insights into a patient’s physical condition.

The metrics derived from wearable devices are referred to as digital biomarkers (dBM), defined as “objective, quantifiable, physiological, and behavioral measures that are collected by means of digital devices that are portable, wearable, implantable, or digestible” [6]. The use of dBM facilitates the continuous measurement of objective data at an individual level in a home environment. This reduces the frequency of hospital visits, allows for ongoing monitoring of a patient’s condition, and enables symptom evaluation based on objective data rather than subjective inputs such as questionnaires. The collection of this data was previously limited to snapshots obtained during in-clinic measurements. Here, the most commonly used sensors for measuring dBM related to physical activity in clinical trials are accelerometers [7].

Despite the potential of HAR and dBM, high-frequency data acquisition imposes several challenges. Large data volumes increase power consumption, data processing time, and storage requirements, which in turn limit the battery life and usability of wearable devices for long-term monitoring [8,9]. Moreover, to support high-frequency sensing, wearable devices may require more sophisticated components, resulting in increased size and cost. For wearable devices to be truly in clinical practice, it is essential to reduce the data volume without compromising the recognition accuracy of key activities.

A key approach to reducing data volume is lowering the sampling frequency. While most commercial devices are used at high frequencies [10,11], previous studies have shown that activity recognition may remain accurate at considerably lower frequencies [12,13]. However, few studies have systematically explored this trade-off in the context of activities particularly relevant to clinical diagnosis and disease severity assessment.

This study aims to determine the minimum sampling frequency required to maintain HAR accuracy for clinically meaningful activities. We focus on two sensor locations—non-dominant wrist and chest—based on their proven performance in previous work [5]. By clarifying the relationship between sampling frequency and recognition accuracy, our findings contribute to the development of more efficient and patient-friendly wearable devices suitable for real-world clinical monitoring.

## 2. Related Work

To contextualize our study, we conducted a targeted literature review focused on the relationship between sampling frequency and HAR performance. Articles were identified through keyword-based searches in Google Scholar using combinations of the terms: “human activity recognition”, “sampling frequency”, “down sampling”, “wearable devices”, and “clinical application”. We included studies published in English that employed wearable sensors and reported on the impact of sampling rate on activity recognition performance. No specific publication date range was applied during the search. Review articles and non-experimental papers were excluded. The most relevant information from the selected related works is summarized in Table 1, including their key objects, methods, and relevance to our clinical focus.

Several studies have investigated the relationship between the sampling frequency and HAR accuracy. One study suggested that basic activities (sleeping, walking, running, cycling, office work, resting, and being active) could be classified with a sampling frequency as low as 0.0166 Hz (1/60 Hz) under specific assumptions such as a minimum activity duration of at least one minute, and the focus on posture-related (e.g., absolute acceleration forces) and inter-frame features (e.g., angle between vectors) [14]. Another study reduced the sampling frequency from 80 Hz for activity classification (sedentary, household, walking, and running), and found no significant difference in recognition accuracy down to 10 Hz [15]. Another study showed that low sampling frequencies (5–10 Hz) could maintain high accuracy in heart rate estimation and activity recognition (walking on a treadmill, running on a treadmill, high-resistance exercise bike, and low-resistance exercise bike), using a photoplethysmography (PPG) wrist-worn sensor with convolutional neural networks (CNNs) and transfer learning [16]. Another study investigated the impact of sampling rates on IMU-based orientation estimation and suggested that the sufficient IMU sampling rate for walking is 100 Hz, running is 200 Hz, and high-speed cyclic movements is 400 Hz [17]. Another study concluded that a sampling frequency of 20 Hz is sufficient for fall detection [18]. Additionally, a study on animals examined how the sampling frequency affects the accuracy of activity classification (swim, rest, burst, chafe, and headshake) and showed that a low sampling frequency of 5 Hz could effectively classify activities [19]. These studies suggest that reducing the sampling frequency to a certain extent does not compromise the accuracy of activity recognition. However, to the best of our knowledge, no studies have explored the relationship between reduced sampling frequency and recognition accuracy for activities associated with diseases, such as COPD or arrhythmia.

The long-term objective of this study was to achieve a simple and objective severity assessment or diagnosis by acquiring and processing the minimum necessary acceleration data using wearable devices. The short-term objective was to determine the extent to which the sampling frequency can be reduced while maintaining recognition accuracy for each activity. Because this was a pilot study, data from healthy individuals were used.

**Table 1:** Summary of the most relevant information from the selected related works.

Study	Target Activities	Sampling Frequencies	Classifier	Main Findings	Relevance to Clinical HAR
Khan et al. [12]	General daily activities (5 benchmark datasets + 4 subjects)	4–250 Hz	SVM ^a^	12–63 Hz sufficient	Relevance to physical activity monitoring
Allik et al. [13]	Static, low intensity, moderate intensity, rhythmical intensity, walking, running, outdoor cycling	13–50 Hz	Decision tree	13 Hz sufficient for most activities except for outdoor cycling	Relevance to physical activity monitoring
Bieber et al. [14]	Sleeping, walking, running, cycling, office work, resting, being active	0.0166 Hz (1/60 Hz)	Decision tree	Classified at 0.0166 Hz (1/60 Hz)	Extremely low sampling rate possible for HAR
Zhang et al. [15]	Sedentary, household, walking, running	5–80 Hz	Logistic regression, decision tree, SVM	10 Hz maintain high accuracy	Relevance to physical activity monitoring
Brophy et al. [16]	Walking, running, high resistance exercise bike, low resistance exercise bike	1–256 Hz	CNNs ^b^	5–10 Hz maintain high accuracy	Relevance to physical activity monitoring
Fan et al. [17]	Walking, running, high-speed cyclic movements	10–1600 Hz	4 SFAs ^c^: FSM ^d^, ECF ^e^, VQF ^f^, SEL ^g^	100 Hz sufficient for walking, 200 Hz running, 400 Hz high-speed cyclic movements	Orientation estimation
Antonio Santoyo-Ramón et al. [18]	Activities of daily living (ADL), fall (15 public datasets)	1–238 Hz	CNNs	20 Hz sufficient for fall detection	Fall detection

^a^ SVM: support vector machine. ^b^ CNNs: convolutional neural networks. ^c^ SFAs: sensor fusion algorithms. ^d^ FSM: finite state machine. ^e^ ECF: extended complementary filter. ^f^ VQF: versatile quaternion-based filter. ^g^ SEL: SFA that eliminates the effect of magnetic disturbances on attitude estimates.

## 3. Materials and Methods

### 3.1. Participants

This study was conducted in accordance with the principles of the Declaration of Helsinki and approved by the Ethics Committee of Okayama University (approval number: R2203-001, issued on 14 April 2022). Written informed consent was obtained from 30 healthy participants (13 males, 17 females) with a mean age of 21.0 ± 0.87 years (range: 19–23 years), and all but one of the participants were right-handed. The participants were recruited through university posts and announcements. Individuals with cardiovascular or respiratory conditions that could pose risks during exercise stress, and pregnant women, were excluded from the study (Table S1). There were no participants who were significantly overweight or underweight. Therefore, we did not record body weight, as we judged that it would not substantially affect the interpretation of the results.

### 3.2. Experimental Setup

**Figure 1:**
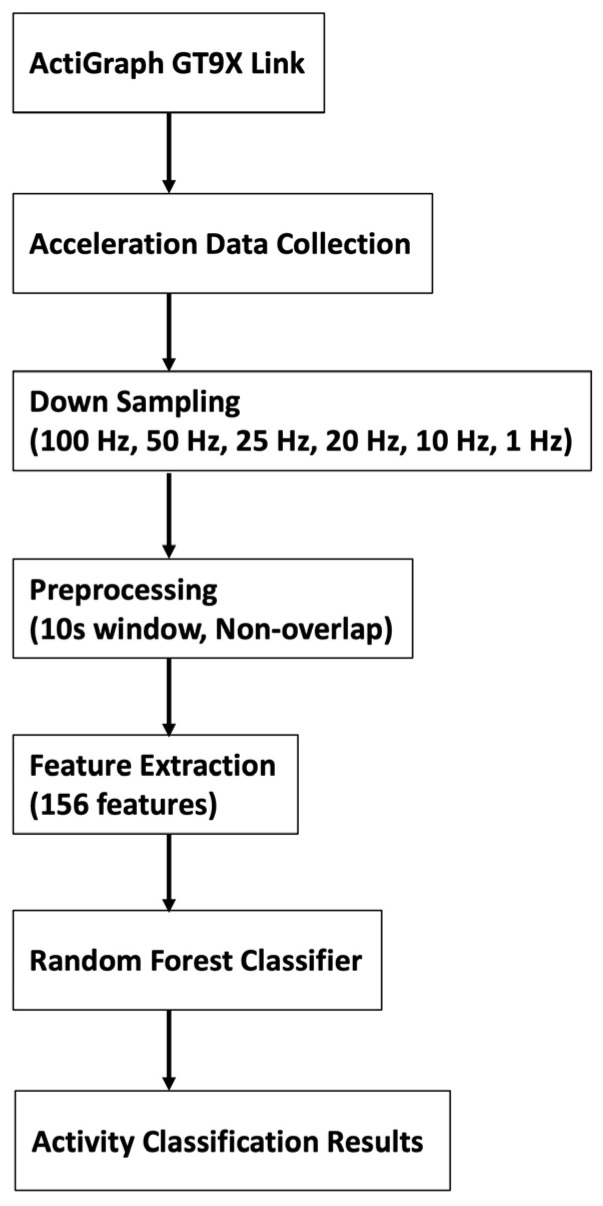
Experimental procedure and data analysis.

Figure 1 describes the experimental procedure and data analysis. The participants wore five 9-axis accelerometers (ActiGraph GT9X Link, ActiGraph LLC, Pensacola, FL, USA) positioned as follows: on the dominant wrist, non-dominant wrist, chest, hip (opposite the dominant hand), and thigh (opposite the dominant hand). In this study, however, we analyzed the data for only two attachment sites, the non-dominant wrist and the chest, which were highly accurate for behavior identification in our previous study [5]. All the devices were configured to a sampling frequency of 100 Hz, synchronized, and had their idle sleep mode disabled.

Participants performed nine activities according to a protocol outlined previously [5]. Briefly, the activities were conducted in the following order: lying in the supine position, standing, sitting, eating, brushing teeth, using the restroom, walking, ascending and descending stairs, and running. Each activity lasted for 2 min and was performed at the participant’s own pace. The interval between eating and brushing teeth, which did not overlap with the other nine activities, was classified as “other movements”. Activity duration was measured using a stopwatch. The recorded times were synchronized with the sensor data by aligning the stopwatch timestamps with the corresponding data points, ensuring accurate activity labeling within the dataset.

### 3.3. Activity Selection

The nine activities were selected based on two criteria: (1) basic activities (lying in the supine position, standing, sitting, walking, ascending or descending stairs, and running). previously recognized using tri-axial or 9-axis accelerometer sensors [20,21]; (2) daily activities reported by COPD patients that caused breathlessness in questionnaires [22] or those commonly documented in Holter ECG monitoring records (eating, brushing teeth, and using the restroom). Detailed explanations of this methodology are provided in our previous study [5].

### 3.4. Data Processing and Feature Extraction

Data were processed using the ActiGraph ActiLife software (version 6.13.4, ActiGraph LLC, Pensacola, FL, USA), following the methodology outlined in a previous study [21]. The extracted data were divided into 10 s non-overlapping segments, from which time- and frequency-domain features were derived [23]. These features included the mean, standard deviation, variance, maximum, minimum, root mean square, signal magnitude area, inter-axis correlation, entropy, energy, kurtosis, skewness, median, interquartile range, and autoregressive coefficients. A total of 156 features per window were used for model training and testing.

### 3.5. Sampling Frequency

Six sampling frequencies were assessed:

(i) At 100 Hz: Original sampling frequency during data collection.
(ii) At 50 Hz: Every second data point in the 100 Hz dataset.
(iii) At 25 Hz: Every second data point in the 50 Hz dataset.
(iv) At 20 Hz: Every fifth data point in the 100 Hz dataset.
(v) At 10 Hz: Every second data point in the 20 Hz dataset.
(vi) At 1 Hz: Every hundredth data point in the 100 Hz dataset.

These downsampled datasets were used to analyze sampling frequency effects.

One methodological consideration is the potential impact of aliasing during downsampling. In this study, we did not apply an anti-aliasing (low-pass) filter prior to downsampling. However, the classification accuracy remained stable even when the sampling frequency was reduced to 10 Hz. This suggests that high-frequency components did not significantly corrupt the low-frequency content required for activity recognition. Therefore, it is unlikely that aliasing artifacts meaningfully influenced the observed results, and additional filtering was deemed unnecessary in the current analysis.

### 3.6. Model Training and Testing

Training and testing followed the protocols of our previous study [5], employing leave-one-subject-out (LOSO) cross-validation [24]. The dataset (30 participants) was divided into a training set (*n* = 29) and a test set (*n* = 1). A random forest (RF) classifier [25] implemented in scikit-learn was trained. RF offers several advantages that make it well-suited for our application: (1) it is relatively easy to interpret and implement, even for researchers new to machine learning, due to its reliance on decision trees; (2) it is a flexible algorithm capable of handling both regression and classification problems, and can manage high-dimensional data with complex, non-linear relationships and interactions among variables; and (3) it includes a built-in feature importance mechanism, allowing for an assessment of which input variables contribute most to model predictions—thereby offering additional interpretability and practical insight for clinical applications [26]. Because of these strengths, RF has been widely used in fields such as healthcare, finance, and marketing. In particular, RF has shown stable and high accuracy in human activity recognition [27,28], making it a suitable choice for the objectives of our study. The following hyperparameters of RF were used: n_estimators = 2500; criterion = “gini”; max_depth = 30; min_samples_split = 2; min_samples_leaf = 1; random_state = 42. The n_estimators and max_depth values were increased stepwise from the default (100 and none, respectively) until recognition accuracy stabilized, indicating convergence in performance. Random_state was set to 42 for reproducibility (default: none); the other parameters remained at their default values. Performance was tested on the holdout participants and the process was repeated 30 times across all participant combinations.

### 3.7. Performance Evaluation

The classifier’s performance was evaluated using the following metrics:

- *Precision*: The proportion of predicted positive cases that were correctly identified, calculated as follows:

Precision = (True positive)/(True positive + False positive)(1)

- *Recall*: The proportion of actual positive cases correctly identified, calculated as follows:

Recall = (True positive)/(True positive + False negative)(2)

- *F-value*: The harmonic mean of precision and recall, calculated as follows:

F-value = 2 × (Precision × Recall)/(Precision + Recall)(3)

Activities with F-values ≥ 0.7 were deemed recognizable, reflecting high-to-moderate accuracy (area under the curve (AUC) ≥ 0.7) as per prior studies [29,30,31]. The confusion matrices supplemented the prediction analyses.

### 3.8. Sensor Placement

GT9X Link devices were placed on the dominant wrist, non-dominant wrist, chest, hip, and thigh. However, because data from the non-dominant wrist and chest have been shown to achieve superior accuracy in activity recognition compared to other body locations [5], we tested two classifiers: one using non-dominant wrist data and the other using chest data. These evaluations evaluated the capacity of the system to detect activities at these sites.

## 4. Results

### 4.1. Waveform Data

**Figure 2:**
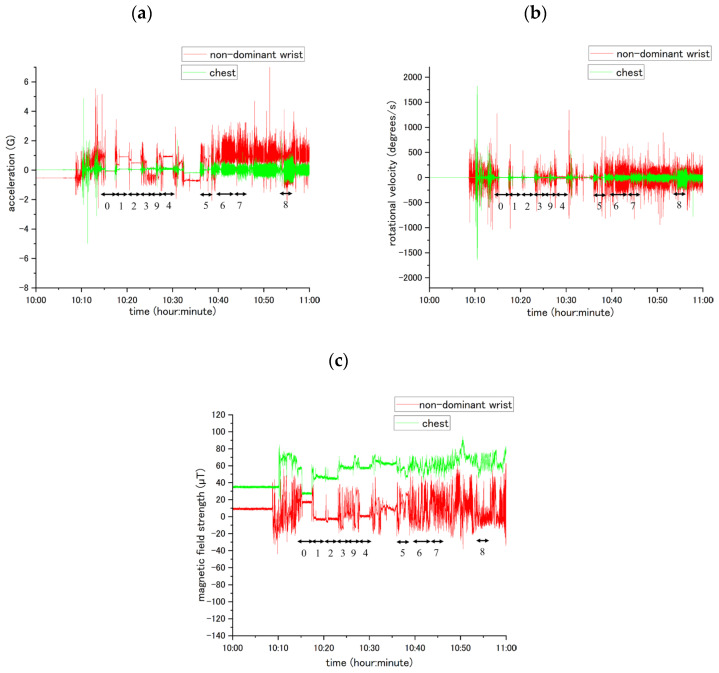
Waveform data from sensors at two body locations: the non-dominant wrist (red line) and the chest (green line). Labels (0–9) indicate activities: 0 = lying in the supine position, 1 = standing, 2 = sitting, 3 = eating, 4 = brushing teeth, 5 = using the restroom, 6 = walking, 7 = ascending/descending the stairs, 8 = running, 9 = other movements. (**a**) X-axis acceleration; (**b**) X-axis angular velocity; (**c**) X-axis magnetic field intensity.

Figure 2, Figures S1 and S2 present typical unprocessed data samples from the x-, y-, and z-axes, respectively, encompassing the acceleration, angular velocity, and magnetic field strength. During the experiments, the participant activities were recorded and synchronized with accelerometer data labeled numerically (0–9). Differences in activity types and sensor locations resulted in unique patterns of variation in acceleration, angular velocity, and magnetic field strength.

### 4.2. Sampling Frequency and Activity Recognition Results

#### 4.2.1. Non-Dominant Wrist Classifier

**Figure 3:**
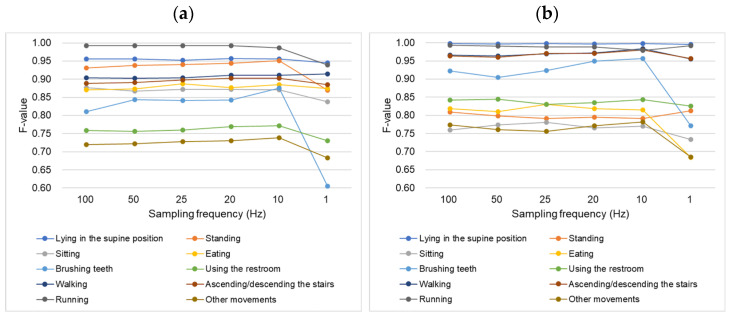
F-value comparison across sampling frequencies for each activity. (**a**) Non-dominant wrist sensor; (**b**) Chest sensor.

The recognition accuracy for various activities was assessed using acceleration data from a nine-axis accelerometer. Activities were considered recognizable if their F-score exceeded 0.7. Table 2a and Figure 3a depict the relationship between the sampling frequency and the activity recognition performance of the non-dominant wrist sensor. For most activities, except for brushing teeth, reducing the sampling frequency to 10 Hz had a minimal impact on recognition accuracy. Except for sitting, all activities achieved their highest F-scores at frequencies below 100 Hz: eating at 25 Hz; lying in the supine position, ascending or descending stairs at 20 Hz; standing, brushing teeth, using the restroom, and other movements at 10 Hz; and walking at 1 Hz. Running maintained consistent F-scores at 100, 50, 25, and 20 Hz. Unlike other activities, brushing teeth exhibited remarkable F-score increases when the frequency decreased from 100 to 50 Hz and from 20 to 10 Hz. However, lowering the frequency to 1 Hz reduced the accuracy for all activities, except walking, which peaked at 1 Hz. Brushing teeth showed a notably larger F-score decline between 10 Hz and 1 Hz compared to other activities, with recognition accuracy decreasing substantially at 1 Hz.

#### 4.2.2. Chest Classifier

Table 2b and Figure 3b depict the relationship between the sampling frequency and activity recognition performance of the chest sensor. For most activities, except for brushing teeth, reducing the sampling frequency to 10 Hz had a minimal impact on the recognition accuracy. Except for lying in the supine position and running, all activities achieved their highest F-scores at frequencies below 100 Hz: using the restroom at 50 Hz, sitting and eating at 25 Hz, brushing teeth, walking, ascending and descending stairs, other movements at 10 Hz, and standing at 1 Hz. Brushing teeth exhibited a remarkable increase in accuracy as the frequency decreased from 50 Hz to 10 Hz, peaking at 10 Hz among the tested frequencies. However, the F-score drop between 10 and 1 Hz for brushing teeth was substantially larger than that for other activities, and its accuracy decreased markedly at 1 Hz, which is consistent with the non-dominant wrist results. Lowering the frequency to 1 Hz notably reduced the accuracy for all activities except for lying in the supine, standing, and running positions. Accuracy for lying in the supine position remained nearly unchanged between 10 Hz and 1 Hz, whereas standing and running showed improved accuracy at 1 Hz compared with 10 Hz.

### 4.3. Confusion Matrices

**Figure 4:**
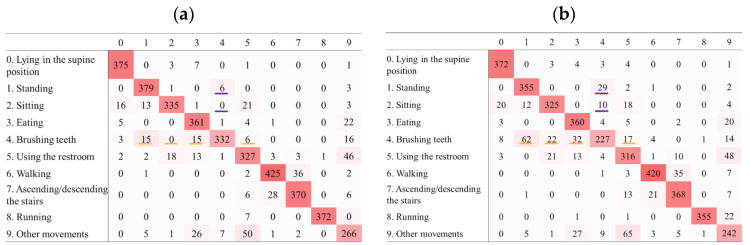
Confusion matrices of predicted versus actual activities using data from the non-dominant wrist sensor. Rows show actual activities; columns show classifier predictions: (**a**) 10 Hz; (**b**) 1 Hz. Orange highlights indicate misclassifications where brushing teeth was mistaken for standing, sitting, eating, or using the restroom. Purple highlights mark errors where standing or sitting were misidentified as brushing teeth. Reducing the sampling frequency from 10 Hz to 1 Hz markedly increases these misclassification errors.

**Figure 5:**
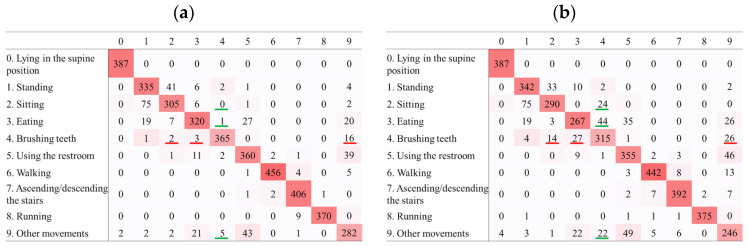
Confusion matrices of predicted versus actual activities using data from the chest sensor. Rows show actual activities; columns show classifier predictions: (**a**) 10 Hz; (**b**) 1 Hz. Red highlights indicate misclassifications where brushing teeth was mistaken for sitting, eating, or other movements. Green highlights mark errors where sitting, eating, or other movements were misidentified as brushing teeth. Reducing the sampling frequency from 10 Hz to 1 Hz markedly increases these misclassification errors.

Figure 4 and Figure 5 show excerpts from the confusion matrices for activity recognition using data from the non-dominant wrist and chest sensors, respectively. The diagonal elements of the matrices indicate that most activities were correctly classified. However, some misclassifications occurred, with patterns varying with sensor location and sampling frequency. The full confusion matrices for all sampling frequencies are shown in Supplementary Figures S3 and S4.

## 5. Discussion

This study is the first to demonstrate that HAR for activities associated with disease conditions can maintain a high accuracy even when the sampling frequency is reduced to 10 Hz. While a 10 Hz sampling frequency preserves recognition accuracy, a further reduction to 1 Hz leads to a decrease in recognition performance across various activities, with brushing teeth showing a notably substantial decline. Among all the activities evaluated, brushing teeth was the most sensitive to sampling frequency variations. The ability to lower the sampling frequency to 10 Hz without compromising accuracy is crucial because it directly addresses one of the primary challenges in long-term patient monitoring: data volume. By reducing the sampling frequency, we effectively reduced the amount of data collected, enabling extended periods of continuous monitoring. This study builds upon our previous work [5] by introducing two key methodological advancements. First, we applied a downsampling approach to systematically examine how changes in sampling frequency affect activity recognition accuracy. Second, instead of using data from five sensor placements as in the earlier study, we focused our analysis on the non-dominant wrist and chest, sites previously identified as yielding the highest classification performance. This advancement is particularly valuable in clinical environments where prolonged patient observation is often necessary but has been limited by data storage and processing constraints. This finding enables more accurate, objective, and practical assessments of disease severity and diagnosis in real-world clinical settings.

Regarding data from the non-dominant wrist, most activities achieved optimal recognition accuracy at sampling frequencies other than 100 Hz. Reducing the sampling frequency from 100 to 10 Hz resulted in no substantial difference in accuracy. In a previous study that used the ActiGraph GT3X+ worn on the hip, the sampling frequency was reduced from 100 Hz. A 10 s window at 50 Hz yielded the highest accuracy with no substantial loss in accuracy observed between 100 and 25 Hz [32]. The finding that reducing the sampling frequency to a certain extent does not impact the accuracy aligns with the results of the present study.

Similarly, for activities other than walking, reducing the sampling frequency from 10 Hz to 1 Hz resulted in a clear decline in recognition accuracy. Given that walking involves slow movements, a 1 Hz sampling frequency appeared sufficient to capture the motion, preventing a decrease in accuracy. A previous study achieved high accuracy in recognizing activities (sedentary, household, walking, and running) using data at 10–20 Hz, but the accuracy declined when the frequency was reduced to 5 Hz [15]. This finding supports the results of the present study, in which a decrease in accuracy was observed when the frequency was lowered from 10 to 1 Hz. Another study investigating the optimal frequencies for activity recognition across multiple datasets found that the accuracy peaked within the 12–63 Hz range [12], which is consistent with our results. Here, most activities achieved maximum F-scores at frequencies of 50 Hz or less, with a sharp drop in accuracy when the frequency was reduced from 10 to 1 Hz. Additionally, another study reported a strong correlation in activity recognition (cycling, mixed activity, sit/stand, sleep, vehicle, and walking) between 100 and 25 Hz [33], aligning with our observation that reducing the frequency from 100 to 25 Hz did not considerably affect accuracy.

Reducing the sampling frequency from 10 to 1 Hz substantially decreased the activity recognition accuracy during brushing teeth. This is likely because the arm moves more rapidly and frequently when brushing teeth. The hand and arm exhibited reciprocating motion at approximately 3–7 Hz [34], exceeding the 1 Hz sampling frequency. Although the device was worn on a non-dominant wrist, vibrations from the toothbrush arm were likely transmitted to it, thereby increasing the movement speed. Thus, a frequency of 1 Hz may have been insufficient to capture these rapid motions, causing a notable decrease in accuracy. Short-duration activities (e.g., running) experience lower recognition accuracy at reduced sampling frequencies [35]. A comparison of the confusion matrices for 10 Hz and 1 Hz revealed that lowering the frequency increased the misclassification of brushing teeth as standing, sitting, eating, or using the restroom. Conversely, stationary activities were more frequently misclassified as brushing teeth. In contrast, the misclassifications of walking, ascending/descending stairs, and running were minimal. This suggests that stationary activities are more prone to confusion, whereas movement-based activities provide clearer acceleration changes and aid recognition. At higher sampling frequencies, the device captured subtle vibrations of brushing teeth more effectively. However, at lower frequencies, this became difficult, increasing the confusion with other stationary activities. The most frequent misclassification occurred between brushing teeth and standing, likely because both involve remaining in place with similar non-dominant wrist directions.

Regarding data from the chest sensor, similar to those from the non-dominant wrist, reducing the sampling frequency from 100 Hz to 10 Hz resulted in no substantial difference in accuracy. However, lowering the frequency to 1 Hz led to a decline in accuracy, with brushing teeth exhibiting a more pronounced reduction in accuracy than other activities, which is consistent with findings from the non-dominant wrist. Rapid arm movements associated with brushing teeth may cause subtle and rapid movements in the chest. Consequently, reducing the sampling frequency from 10 Hz to 1 Hz may have made it difficult to capture these fine vibrations, leading to a substantial decrease in recognition accuracy. A comparison of the confusion matrices for 10 Hz and 1 Hz revealed that lowering the frequency from 10 Hz to 1 Hz increased the misclassification of brushing teeth as sitting, eating, or other activities. In addition, sitting, eating, and other activities were more frequently misclassified as brushing teeth. Both sitting and eating are stationary activities performed while seated, and chest movements during brushing teeth also constitute stationary activities. At higher sampling frequencies, the chest vibrations associated with brushing teeth could be captured, but at lower frequencies, detecting these vibrations became challenging, potentially leading to confusion with other stationary activities, such as sitting or eating, where the chest similarly remains in a static state.

Reducing the sampling frequency to 1 Hz resulted in a decline in the recognition accuracy for many activities; however, the accuracy remained largely unaffected when lying in the supine position, standing, and running. In contrast, when measured at the non-dominant wrist, these activities exhibited reduced accuracy at 1 Hz. The non-dominant wrist remains free to move while lying in the supine and standing positions, potentially leading to wrist movements in some individuals. However, while lying in the supine and standing positions, the chest remained stationary, exhibiting less movement than the non-dominant wrist, which may explain why the accuracy did not decrease at 1 Hz. During running, the non-dominant wrist undergoes considerable motion owing to the forward and backward swinging of the arm, whereas the chest primarily experiences translational movement with less rapid motion than the wrist. Consequently, a 1 Hz sampling frequency was sufficient to capture the movements of the chest.

Regardless of whether the device was worn on the non-dominant wrist or chest, the recognition accuracy for all activities targeted in this study was maintained when the sampling frequency was reduced to 10 Hz, regardless of the activity type. For some activities, reducing the frequency to 1 Hz did not affect the recognition accuracy; however, for brushing teeth, a sampling frequency of 10 Hz or higher was essential. Nonetheless, because ECG during brushing teeth exhibits a distinct pattern when implementing an accelerometer in a Holter ECG device, it is not necessary to maintain a 10 Hz sampling frequency solely to preserve brushing teeth recognition accuracy; reducing it to 1 Hz would suffice. The ActiGraph GT9X Link has a maximum sampling frequency of 100 Hz. However, acquiring data at this maximum rate is unnecessary, and reducing it to a minimum configurable frequency of 30 Hz is acceptable. Switching to a nine-axis measurement markedly increases battery consumption, reducing the measurable duration to approximately 1/14 of that achieved with a three-axis measurement [36]. Therefore, lowering the sampling frequency to the maximum possible extent is critical to extend the measurement duration, even if only marginally.

Based on our findings, we offer the following practical recommendations for selecting an appropriate sampling frequency in HAR systems used in clinical settings:

∙ A sampling frequency of 10 Hz is sufficient to maintain reliable recognition accuracy when using data from wearable accelerometers, even in long-term monitoring scenarios.
∙ Reducing the sampling rate from 100 Hz to 10 Hz significantly decreases data volume and power consumption, enabling longer battery life and extended monitoring, which is particularly advantageous for clinical use.
∙ For specific high-frequency, short-duration movements such as brushing teeth, higher sampling rates may improve recognition. However, in the context of Holter ECG monitoring, where brushing teeth can be identified based on ECG waveform patterns, a sampling rate as low as 1 Hz may be acceptable.
∙ Therefore, we suggest 10 Hz as a general lower limit for HAR in clinical applications, with the possibility of further reduction (e.g., to 1 Hz).

Applying the findings of this study to clinical settings may enable simpler and more objective severity assessments and diagnoses. For instance, the severity of COPD can be evaluated by monitoring the activity of patients with COPD using an acceleration sensor and analyzing the extent of SpO_2_ reduction during specific activities. A greater reduction in SpO_2_ during activities associated with shortness of breath could indicate a higher severity. Additionally, recognizing the activities of patients wearing a Holter ECG device during episodes of palpitations or shortness of breath eliminates the need for patients to manually record their activities, thereby facilitating an objective arrhythmia diagnosis using accelerometer data. Traditionally, Holter ECG measurement duration has been limited to 24 h. However, in recent years, devices capable of long-term monitoring (beyond 24 h) have been developed to detect conditions such as paroxysmal atrial fibrillation (PAF) and asymptomatic arrhythmias that are difficult to capture within a 24 h window [37,38]. In a 24 h Holter ECG examination, the detection rate of arrhythmias is 30%, whereas extending the monitoring period to 1 week increases the detection rate to 90% [39]. Consequently, reducing the sampling frequency to minimize the impact on battery life is becoming increasingly critical.

Activity recognition was conducted as a pilot study using healthy individuals as participants. The targeted activities included those known to cause shortness of breath in patients with COPD and those listed on the record card of a Holter ECG monitor. Based on the results of this study, the next phase will involve collecting data from patients.

Continuous data collection via wearable devices enables the real-time monitoring of a patient’s condition, supporting the provision of personalized medical care. In particular, wearable-derived indicators can reflect subtle physiological or behavioral changes over time that may not be captured during periodic clinical visits. If such indicators are incorporated into routine clinical assessments alongside traditional biomarkers, they may enable a more nuanced and individualized evaluation of disease status. Moreover, long-term wearable data could facilitate the early detection of symptom exacerbations or disease progression, reducing the need for frequent hospital visits and improving patient outcomes. These developments highlight the potential of wearable-based monitoring to serve as a foundation for data-driven, personalized care in chronic disease management.

In real-world clinical environments, several practical obstacles would be encountered when using the nine-axis system. One of the major challenges is ensuring that patients consistently wear the device correctly. Misalignment or improper placement could compromise the accuracy of sensor data. Maintaining continuous wear over extended periods is also difficult, especially among elderly patients or those with cognitive impairments. Some participants might remove the device unintentionally, while others report discomfort or skin irritation, raising concerns about user compliance and safety. From a technical perspective, sensor noise and signal drift pose challenges to long-term accuracy. In addition, magnetometer readings are occasionally affected by environmental interference (e.g., metallic objects or medical equipment), which could impact motion tracking reliability [40]. These issues underscore the importance of robust sensor calibration, user training, and thoughtful device design for clinical deployment.

Scaling up the use of nine-axis sensor technology in clinical practice poses both opportunities and challenges. From a cost perspective, equipping a large patient population with such devices would require significant investment not only in hardware, but also in maintenance, replacement, and data infrastructure. Additionally, ensuring consistent and correct wear by patients becomes more difficult at scale. Clinical teams would need to implement education, training, and monitoring strategies to maintain data quality. The risk of device loss or damage also increases with broader deployment, especially in home-based or long-term monitoring scenarios. Despite these practical barriers, the clinical benefits, particularly for objective, long-term activity monitoring and symptom-context association (e.g., in COPD or arrhythmia), may justify the investment.

This study has some limitations. First, the participants were young and healthy, which may not adequately reflect the movement patterns of patients with COPD or other chronic conditions. This limitation is particularly relevant when considering that individual characteristics, such as age and mobility limitations, can significantly influence the impact of sampling frequency on HAR model performance. For example, in elderly individuals, walking and running may produce similar motion patterns due to reduced gait speed and limited acceleration range. In such cases, a higher sampling frequency (e.g., 20–50 Hz) may be required to capture subtle differences in periodicity, amplitude, and transition dynamics between these closely related activities. Lower sampling frequencies may lack sufficient resolution to distinguish them accurately. A previous study recognized gait patterns in older adults using wearable smartwatch devices at a sampling frequency of 50 Hz [41]. Future studies should include older adults and clinical populations to validate the model in relevant user groups. Second, while a LOSO cross-validation method was utilized, additional testing under noisy or missing data conditions is necessary to improve the system’s robustness for real-world clinical environments. Techniques such as data augmentation [42] or denoising autoencoders [43] could be explored to address this issue. Third, the activities were conducted in controlled settings, which may not fully replicate real-world conditions, potentially leading to reduced activity recognition accuracy in practical applications. Future work should incorporate data collected in uncontrolled, real-life environments, such as during free-living activities at home or in a clinical ward.

## 6. Conclusions

This study investigated the effects of reducing the sampling frequency on HAR accuracy using machine learning, to optimize data collection for clinical applications. The key contributions of this study are as follows:

(1) Reducing the sampling frequency to 10 Hz did not considerably affect the recognition accuracy for most activities when using data from either the non-dominant wrist or chest sensors.
(2) Further reduction to 1 Hz resulted in decreased accuracy for many activities, with a decline in the recognition of brushing teeth.
(3) The ability to maintain recognition accuracy at a sampling frequency of 10 Hz has important implications for clinical applications, allowing for a substantial reduction in data volume, enabling long-term patient monitoring. A reduced data volume can lead to extended battery life, faster data processing and transmission, and potential device miniaturization. These improvements have made wearable devices suitable for continuous long-term monitoring in clinical settings.
(4) The study demonstrated the feasibility of using lower sampling frequencies for HAR in clinical applications, particularly for conditions where the relationship between symptom onset and patient activity is crucial, such as COPD and arrhythmia. This study provides valuable insights for the development of more efficient and patient-friendly wearable devices for clinical monitoring. Optimizing the sampling frequency will pave the way for improved long-term patient monitoring, and potentially more objective and simplified diagnostic approaches for various medical conditions.

## Figures and Tables

**Table 2:** (**a**) Performance evaluation of the non-dominant wrist classifier. The highest F-values are shown in bold. (**b**) Performance evaluation of the chest classifier. The highest F-values are shown in bold.

(a)				
	Sampling Frequency (Hz)	Precision	Recall	F-Value
Lying in the supine position	100	0.9446	0.9737	0.9552
	50	0.9452	0.9763	0.9563
	25	0.9454	0.9686	0.9527
	20	0.9480	0.9737	**0.9567**
	10	0.9509	0.9686	0.9559
	1	0.9411	0.9611	0.9454
Standing	100	0.9350	0.9459	0.9306
	50	0.9351	0.9588	0.9383
	25	0.9372	0.9588	0.9406
	20	0.9422	0.9615	0.9438
	10	0.9413	0.9744	**0.9511**
	1	0.8622	0.9126	0.8694
Sitting	100	0.9171	0.8690	**0.8769**
	50	0.9150	0.8613	0.8669
	25	0.9185	0.8641	0.8724
	20	0.9189	0.8641	0.8720
	10	0.9233	0.8615	0.8707
	1	0.8866	0.8353	0.8382
Eating	100	0.8650	0.9068	0.8712
	50	0.8695	0.9042	0.8737
	25	0.8751	0.9196	**0.8867**
	20	0.8691	0.9093	0.8765
	10	0.8728	0.9196	0.8852
	1	0.8584	0.9160	0.8746
Brushing teeth	100	0.8433	0.7979	0.8104
	50	0.8889	0.8209	0.8433
	25	0.8810	0.8207	0.8418
	20	0.8816	0.8233	0.8420
	10	0.9275	0.8541	**0.8756**
	1	0.6851	0.5829	0.6053
Using the restroom	100	0.7904	0.7634	0.7588
	50	0.7955	0.7582	0.7560
	25	0.7956	0.7601	0.7597
	20	0.8057	0.7733	0.7694
	10	0.8007	0.7841	**0.7720**
	1	0.7300	0.7555	0.7307
Walking	100	0.9295	0.9010	0.9039
	50	0.9312	0.8999	0.9024
	25	0.9259	0.9071	0.9041
	20	0.9295	0.9126	0.9113
	10	0.9355	0.9137	0.9110
	1	0.9412	0.9049	**0.9150**
Ascending/descending the stairs	100	0.8979	0.8920	0.8890
	50	0.8979	0.8949	0.8905
	25	0.9087	0.8970	0.8982
	20	0.9151	0.8994	**0.9028**
	10	0.9142	0.9017	0.9023
	1	0.8812	0.8991	0.8847
Running	100	0.9974	0.9897	**0.9926**
	50	0.9974	0.9897	**0.9926**
	25	0.9974	0.9897	**0.9926**
	20	0.9974	0.9897	**0.9926**
	10	0.9974	0.9821	0.9864
	1	0.9952	0.9361	0.9394
Other movements	100	0.7316	0.7423	0.7192
	50	0.7315	0.7484	0.7226
	25	0.7329	0.7587	0.7281
	20	0.7380	0.7523	0.7300
	10	0.7520	0.7623	**0.7386**
	1	0.7124	0.6930	0.6826
(**b**)				
	**Sampling Frequency (Hz)**	**Precision**	**Recall**	**F-Value**
Lying in the supine position	100	0.9956	1.0000	**0.9976**
	50	0.9938	1.0000	0.9966
	25	0.9956	1.0000	**0.9976**
	20	0.9938	1.0000	0.9966
	10	0.9956	1.0000	**0.9976**
	1	0.9922	1.0000	0.9956
Standing	100	0.8528	0.8675	0.8094
	50	0.8571	0.8466	0.7984
	25	0.8526	0.8438	0.7913
	20	0.8189	0.8590	0.7942
	10	0.8137	0.8590	0.7917
	1	0.8303	0.8769	**0.8123**
Sitting	100	0.8119	0.7667	0.7591
	50	0.8346	0.7846	0.7737
	25	0.8265	0.7923	**0.7804**
	20	0.8472	0.7795	0.7651
	10	0.8192	0.7846	0.7704
	1	0.7993	0.7462	0.7330
Eating	100	0.8442	0.8161	0.8187
	50	0.8488	0.8008	0.8100
	25	0.8714	0.8195	**0.8302**
	20	0.8551	0.8144	0.8185
	10	0.8525	0.8118	0.8151
	1	0.7625	0.6743	0.6843
Brushing teeth	100	0.9386	0.9171	0.9224
	50	0.9294	0.8989	0.9048
	25	0.9366	0.9220	0.9229
	20	0.9712	0.9353	0.9496
	10	0.9762	0.9429	**0.9564**
	1	0.8086	0.8122	0.7715
Using the restroom	100	0.8567	0.8569	0.8420
	50	0.8569	0.8656	**0.8449**
	25	0.8515	0.8543	0.8306
	20	0.8491	0.8615	0.8346
	10	0.8588	0.8675	0.8433
	1	0.8421	0.8546	0.8258
Walking	100	0.9761	0.9593	0.9661
	50	0.9730	0.9568	0.9632
	25	0.9778	0.9637	0.9694
	20	0.9752	0.9710	0.9717
	10	0.9912	0.9776	**0.9839**
	1	0.9681	0.9466	0.9549
Ascending/descending the stairs	100	0.9532	0.9785	0.9641
	50	0.9501	0.9737	0.9598
	25	0.9619	0.9833	0.9712
	20	0.9657	0.9761	0.9704
	10	0.9734	0.9901	**0.9798**
	1	0.9592	0.9563	0.9563
Running	100	1.0000	0.9889	**0.9933**
	50	1.0000	0.9861	0.9912
	25	1.0000	0.9833	0.9889
	20	1.0000	0.9833	0.9889
	10	0.9976	0.9750	0.9788
	1	0.9951	0.9897	0.9921
Other movements	100	0.7917	0.7777	0.7739
	50	0.7781	0.7660	0.7606
	25	0.7779	0.7621	0.7556
	20	0.7977	0.7722	0.7713
	10	0.7997	0.7890	**0.7817**
	1	0.7189	0.6893	0.6849

## Data Availability

The data presented in this study are available upon request from the corresponding author. The data are not publicly available due to confidentiality issues.

## Supplementary Materials

The following supporting information can be downloaded at: https://www.mdpi.com/article/10.3390/s25123780/s1, Table S1: Participant characteristics. Figure S1: Waveform data from sensors at two body locations: the non-dominant wrist (red line) and the chest (green line). The figure represents the same participant as Figure 1. Labels (0–9) indicate activities: 0 = lying in the supine position, 1 = standing, 2 = sitting, 3 = eating, 4 = brushing teeth, 5 = using the restroom, 6 = walking, 7 = ascending/descending the stairs, 8 = running, 9 = other movements. (a) Y-axis acceleration; (b) Y-axis angular velocity; (c) Y-axis magnetic field intensity. Figure S2: Waveform data from sensors at two body locations: the non-dominant wrist (red line) and the chest (green line). The figure represents the same participant as Figure 1. Labels (0–9) indicate activities: 0 = lying in the supine position, 1 = standing, 2 = sitting, 3 = eating, 4 = brushing teeth, 5 = using the restroom, 6 = walking, 7 = ascending/descending the stairs, 8 = running, 9 = other movements. (a) Z-axis acceleration; (b) Z-axis angular velocity; (c) Z-axis magnetic field intensity. Figure S3: Confusion matrices of predicted versus actual activities using data from the non-dominant wrist sensor. Rows show actual activities; columns show classifier predictions. (a) 100 Hz; (b) 50 Hz; (c) 25 Hz; (d) 20 Hz; (e) 10 Hz; (f) 1 Hz. Figure S4: Confusion matrices of predicted versus actual activities using data from the chest sensor. Rows show actual activities; columns show classifier predictions. (a) 100 Hz; (b) 50 Hz; (c) 25 Hz; (d) 20 Hz; (e) 10 Hz; (f) 1 Hz.

## Abbreviations

The following abbreviations are used in this manuscript:

dBM	Digital biomarker
HAR	Human activity recognition
COPD	Chronic obstructive pulmonary disease
ECG	Electrocardiography
LOSO	Leave-one-subject-out
RF	Random forest
AUC	Area under the curve
PAF	Paroxysmal atrial fibrillation
