# Supplementary material for: Effects of Sampling Frequency on Human Activity Recognition with Machine Learning Aiming at Clinical Applications
Source: Sensors (Basel). 2025 Jun 17;25(12):3780. doi: 10.3390/s25123780 (PMC12196717; doi:10.3390/s25123780)
Supplement: Supplementary file 1 [file sensors-25-03780-s001.zip › sensors-3635055-supplementary Figure.pdf]

Supplementary materials

Figure S1. Waveform data from sensors at two body locations: the non-dominant wrist (red line) and the chest (green line). The figure represents the same participant as Figure 1. Labels (0–9) indicate activities: 0 = lying in the supine position, 1 = standing, 2 = sitting, 3 = eating, 4 = brushing teeth, 5 = using the restroom, 6 = walking, 7 = ascending/descending the stairs, 8 = running, 9 = other movements. (**a**) Y-axis acceleration; (**b**) Y-axis angular velocity; (**c**) Y-axis magnetic field intensity.

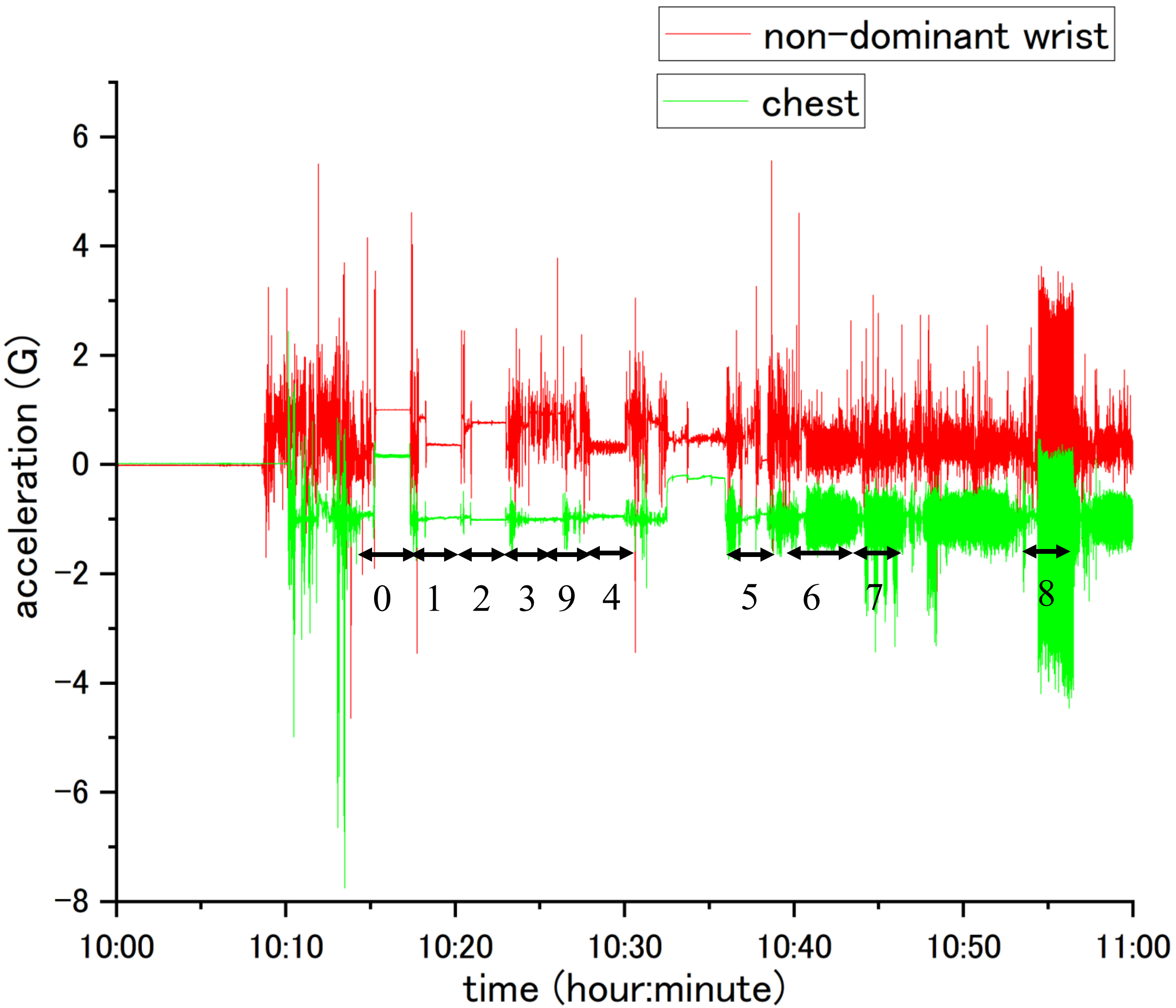
non-dominant wrist
chest
acceleration (G)
6
4
2
0
−2
−4
−6
−8
0
1
2
3
9
4
5
6
7
8
10:00
10:10
10:20
10:30
10:40
10:50
11:00
time (hour:minute)
(a)

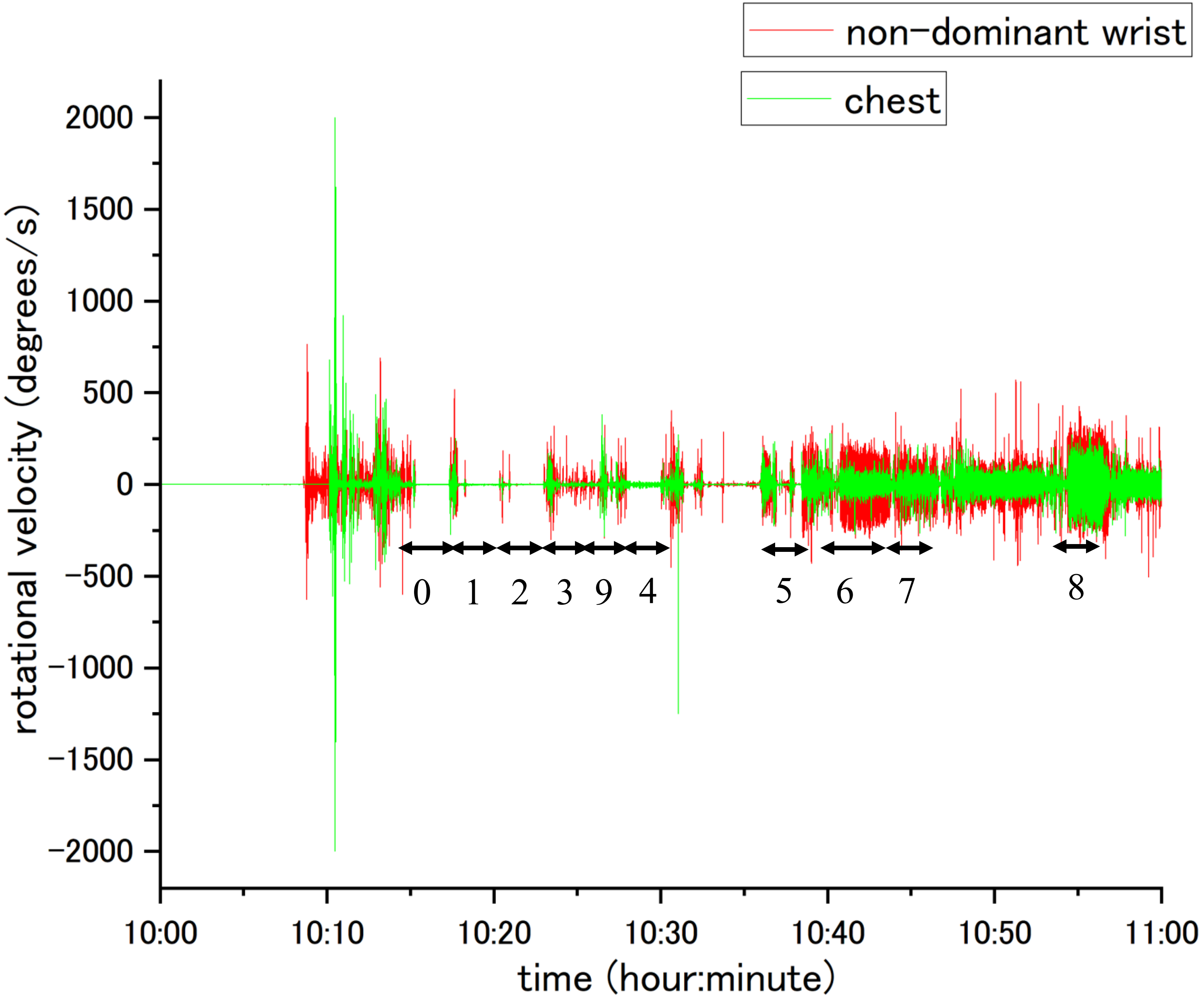

non-dominant wrist
chest
rotational velocity (degrees/s)
2000
1500
1000
500
0
−500
−1000
−1500
−2000
0
1
2
3
9
4
5
6
7
8
10:00
10:10
10:20
10:30
10:40
10:50
11:00
time (hour:minute)

(b)

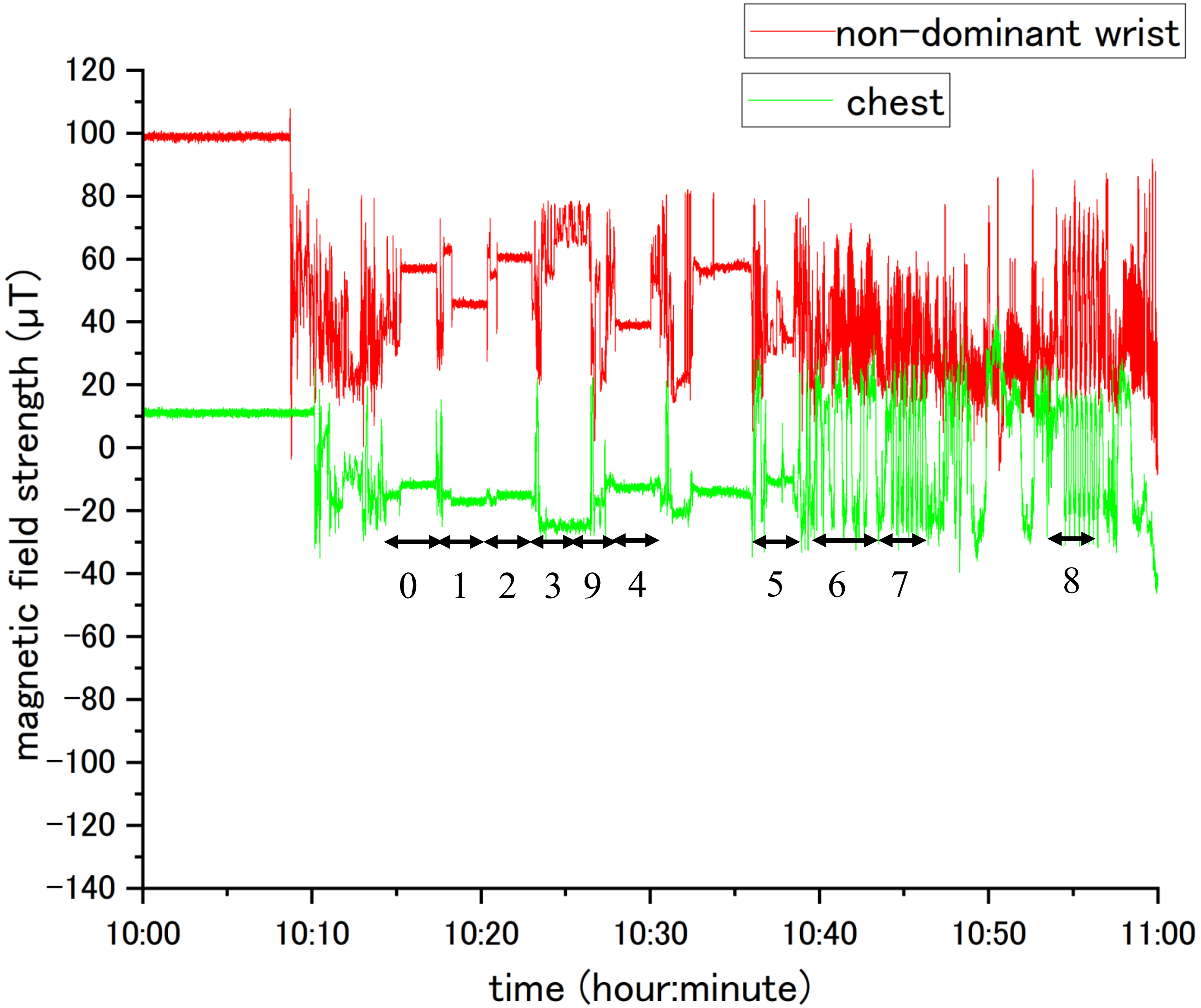
non-dominant wrist
chest
magnetic field strength (μT)
time (hour:minute)
120
100
80
60
40
20
0
−20
−40
−60
−80
−100
−120
−140
10:00
10:10
10:20
10:30
10:40
10:50
11:00
0
1
2
3
9
4
5
6
7
8

(c)

Supplementary materials

Figure S2. Waveform data from sensors at two body locations: the non-dominant wrist (red line) and the chest (green line). The figure represents the same participant as Figure 1. Labels (0–9) indicate activities: 0 = lying in the supine position, 1 = standing, 2 = sitting, 3 = eating, 4 = brushing teeth, 5 = using the restroom, 6 = walking, 7 = ascending/descending the stairs, 8 = running, 9 = other movements. (**a**) Z-axis acceleration; (**b**) Z-axis angular velocity; (**c**) Z-axis magnetic field intensity.

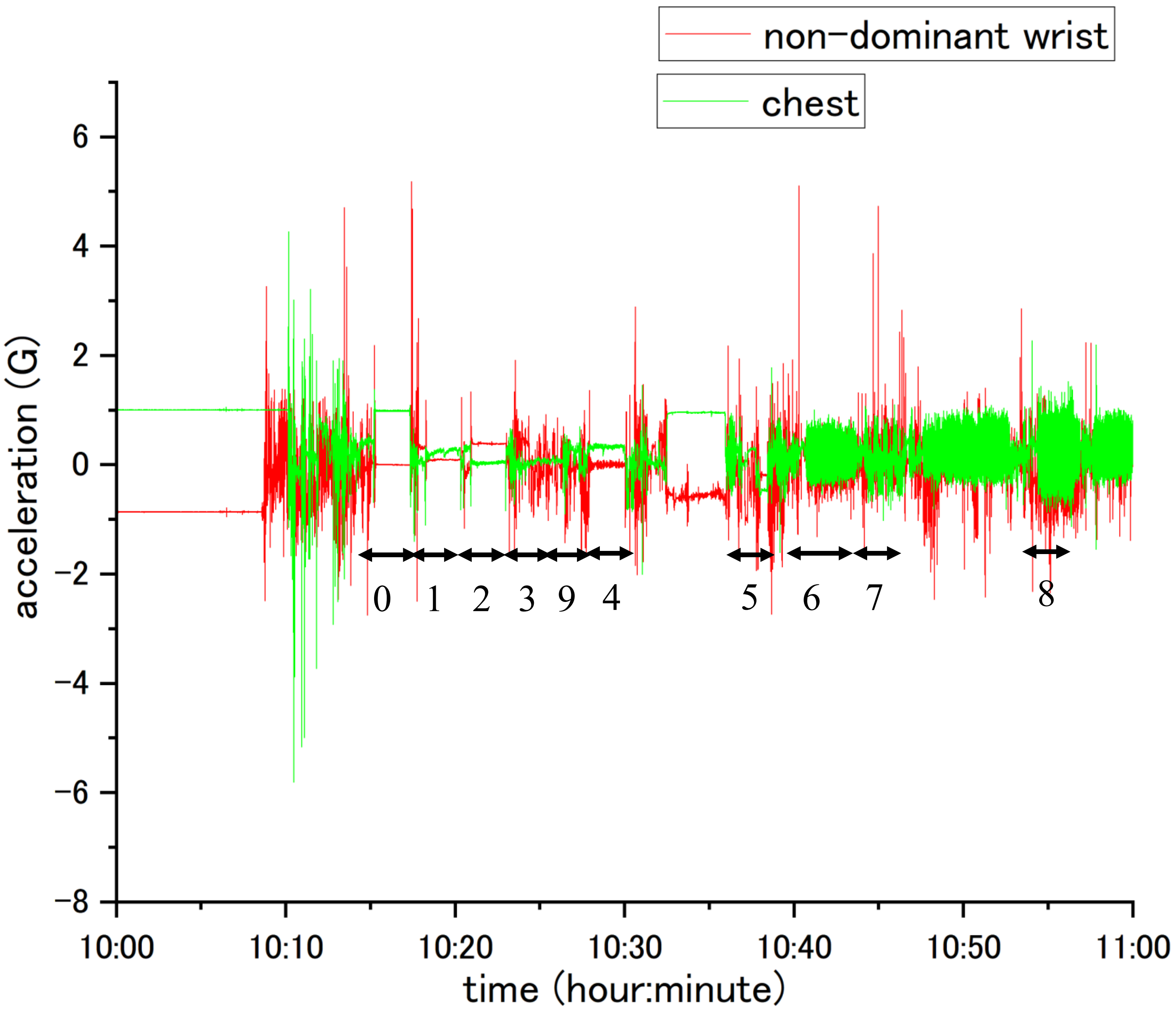
non-dominant wrist
chest
acceleration (G)
6
4
2
0
−2
−4
−6
−8
0
1
2
3
9
4
5
6
7
8
10:00
10:10
10:20
10:30
10:40
10:50
11:00
time (hour:minute)
(a)

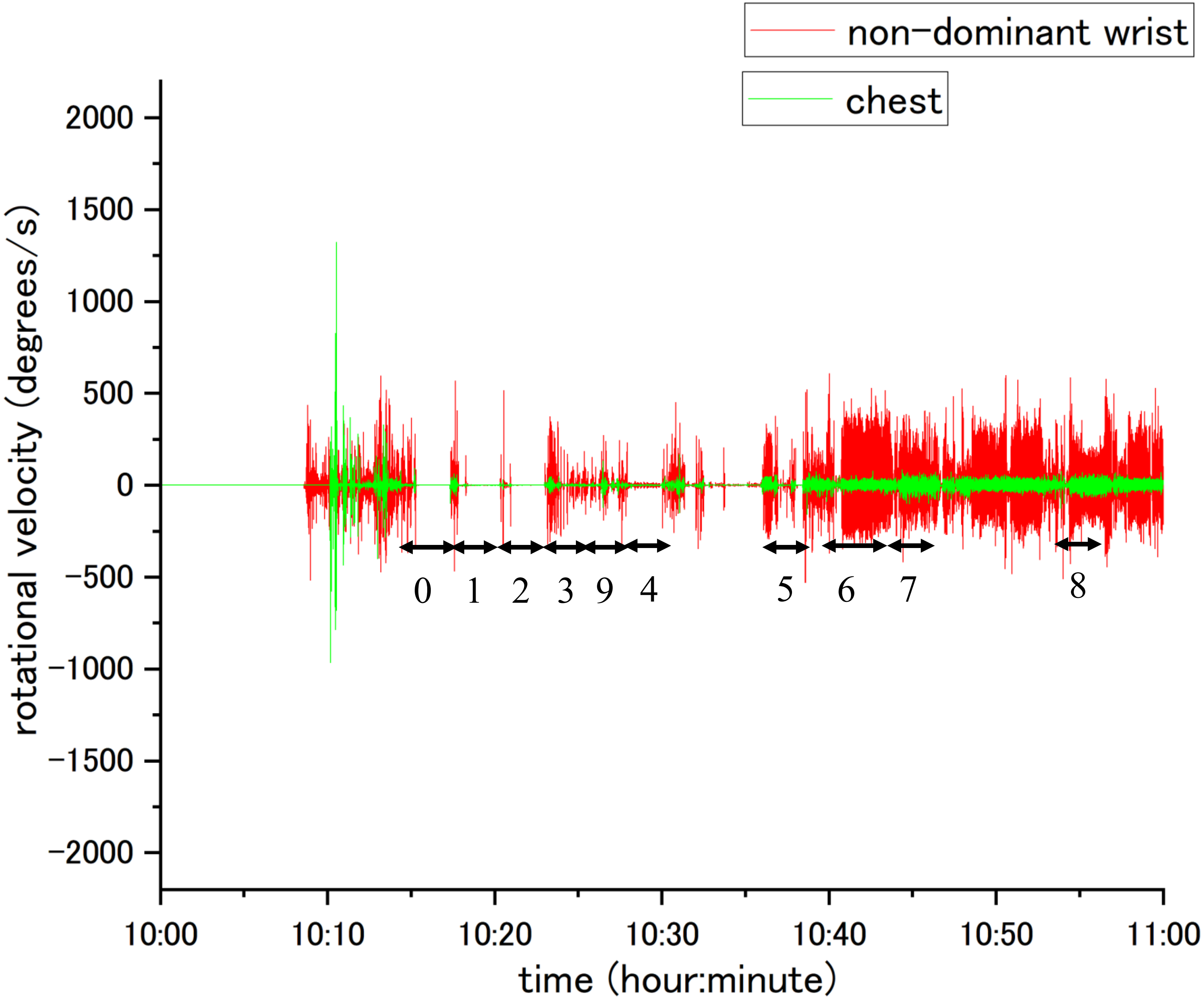
non-dominant wrist
chest
rotational velocity (degrees/s)
2000
1500
1000
500
0
−500
−1000
−1500
−2000
0
1
2
3
9
4
5
6
7
8
10:00
10:10
10:20
10:30
10:40
10:50
11:00
time (hour:minute)
(b)

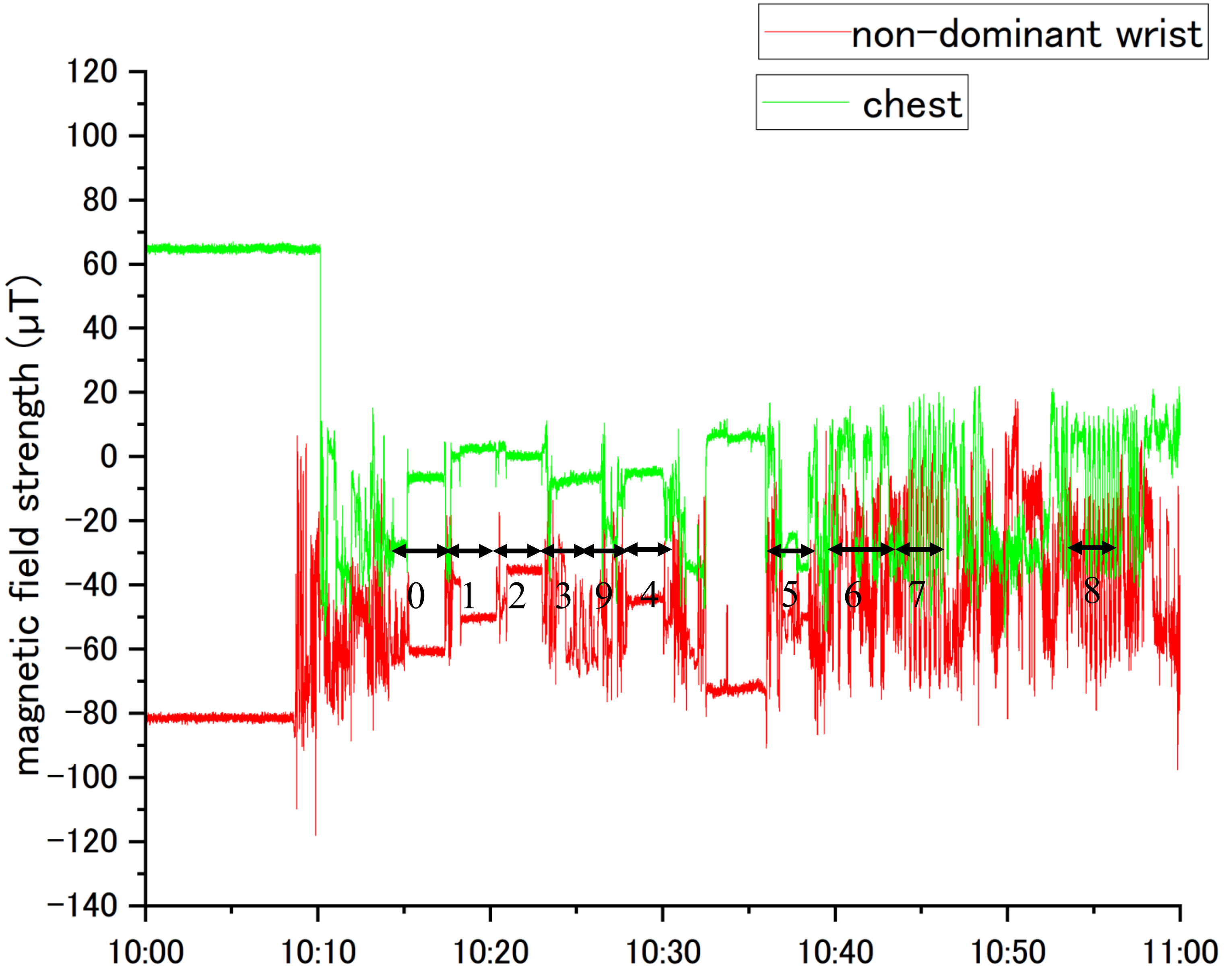
non−dominant wrist
chest
magnetic field strength (μT)
120
100
80
60
40
20
0
−20
−40
−60
−80
−100
−120
−140
0
1
2
3
9
4
5
6
7
8
10:00
10:10
10:20
10:30
10:40
10:50
11:00
time (hour:minute)

(c)

Supplementary materials

Figure S3. Confusion matrices of predicted versus actual activities using data from the non-dominant wrist sensor. Rows show actual activities; columns show classifier predictions. (**a**) 100 Hz; (**b**) 50 Hz; (**c**) 25 Hz; (**d**) 20 Hz; (**e**) 10 Hz; (**f**) 1 Hz.

Non-dominant wrist, 100 Hz

	0	1	2	3	4	5	6	7	8	9
0. Lying in the supine position	377	0	2	6	0	1	0	0	0	1
1. Standing	0	368	1	0	17	0	0	0	0	3
2. Sitting	18	13	338	0	0	17	0	0	0	3
3. Eating	6	0	0	356	3	5	1	0	0	23
4. Brushing teeth	2	21	2	29	310	8	0	0	0	15
5. Using the restroom	3	2	20	12	1	319	3	5	1	50
6. Walking	0	0	0	0	1	2	420	40	0	3
7. Ascending/descending the stairs	0	0	0	0	0	6	31	366	0	7
8. Running	0	0	0	0	0	4	0	0	375	0
9. Other movements	0	3	1	26	13	53	1	1	0	260

(a)

Non-dominant wrist, 50 Hz

	0	1	2	3	4	5	6	7	8	9
0. Lying in the supine position	378	0	2	5	0	1	0	0	0	1
1. Standing	0	373	1	0	13	0	0	0	0	2
2. Sitting	20	13	335	0	0	18	0	0	0	3
3. Eating	6	0	0	355	2	4	1	0	0	26
4. Brushing teeth	2	22	2	21	319	8	0	0	0	13
5. Using the restroom	2	2	20	11	1	317	3	5	1	54
6. Walking	0	0	0	0	1	2	419	41	0	3
7. Ascending/descending the stairs	0	0	0	0	0	6	30	367	0	7
8. Running	0	0	0	0	0	4	0	0	375	0
9. Other movements	0	2	2	27	12	50	1	1	0	263

(b)

Non-dominant wrist, 25 Hz

	0	1	2	3	4	5	6	7	8	9
0. Lying in the supine position	375	0	3	7	0	1	0	0	0	1
1. Standing	0	373	2	0	12	0	0	0	0	2
2. Sitting	18	13	336	0	0	19	0	0	0	3
3. Eating	5	0	0	361	1	4	1	0	0	22
4. Brushing teeth	3	21	2	16	319	7	1	0	0	18
5. Using the restroom	3	2	18	12	1	318	5	2	1	54
6. Walking	0	0	0	0	1	2	422	38	0	3
7. Ascending/descending the stairs	0	0	0	0	0	7	29	368	0	6
8. Running	0	0	0	0	0	4	0	0	375	0
9. Other movements	0	2	1	26	13	46	2	2	0	266

(c)

Non-dominant wrist, 20 Hz

	0	1	2	3	4	5	6	7	8	9
0. Lying in the supine position	377	0	2	6	0	1	0	0	0	1
1. Standing	0	374	1	0	12	0	0	0	0	2
2. Sitting	18	13	336	0	0	20	0	0	0	2
3. Eating	5	0	0	357	2	4	1	0	0	25
4. Brushing teeth	3	19	2	21	320	6	0	0	0	16
5. Using the restroom	2	2	20	12	1	323	5	2	1	48
6. Walking	0	0	0	0	1	2	425	35	0	3
7. Ascending/descending the stairs	0	0	0	0	0	6	28	369	0	7
8. Running	0	0	0	0	0	4	0	0	375	0
9. Other movements	0	2	1	27	13	48	2	1	0	264

(d)

Non-dominant wrist, 10 Hz

	0	1	2	3	4	5	6	7	8	9
0. Lying in the supine position	375	0	3	7	0	1	0	0	0	1
1. Standing	0	379	1	0	6	0	0	0	0	3
2. Sitting	16	13	335	1	0	21	0	0	0	3
3. Eating	5	0	0	361	1	4	1	0	0	22
4. Brushing teeth	3	15	0	15	332	6	0	0	0	16
5. Using the restroom	2	2	18	13	1	327	3	3	1	46
6. Walking	0	1	0	0	0	2	425	36	0	2
7. Ascending/descending the stairs	0	0	0	0	0	6	28	370	0	6
8. Running	0	0	0	0	0	7	0	0	372	0
9. Other movements	0	5	1	26	7	50	1	2	0	266

(e)

Non-dominant wrist, 1 Hz

	0	1	2	3	4	5	6	7	8	9
0. Lying in the supine position	372	0	3	4	3	4	0	0	0	1
1. Standing	0	355	0	0	29	2	1	0	0	2
2. Sitting	20	12	325	0	10	18	0	0	0	4
3. Eating	3	0	0	360	4	5	0	2	0	20
4. Brushing teeth	8	62	22	32	227	17	4	0	1	14
5. Using the restroom	3	0	21	13	4	316	1	10	0	48
6. Walking	0	0	0	0	1	3	420	35	0	7
7. Ascending/descending the stairs	0	1	0	0	0	13	21	368	0	7
8. Running	0	0	0	1	0	1	0	0	355	22
9. Other movements	0	5	1	27	9	65	3	5	1	242

(f)

Supplementary materials

Figure S4. Confusion matrices of predicted versus actual activities using data from the chest sensor. Rows show actual activities; columns show classifier predictions. (**a**) 100 Hz; (**b**) 50 Hz; (**c**) 25 Hz; (**d**) 20 Hz; (**e**) 10 Hz; (**f**) 1 Hz.

Chest, 100 Hz

	0	1	2	3	4	5	6	7	8	9
0. Lying in the supine position	387	0	0	0	0	0	0	0	0	0
1. Standing	0	338	36	7	4	1	0	0	0	3
2. Sitting	0	78	298	8	1	1	1	0	0	2
3. Eating	0	13	7	322	6	31	0	0	0	15
4. Brushing teeth	0	1	3	7	355	2	0	0	0	19
5. Using the restroom	0	0	1	11	1	356	0	4	0	43
6. Walking	0	0	0	0	0	0	448	13	0	5
7. Ascending/descending the stairs	0	0	0	0	0	1	8	401	0	0
8. Running	0	0	0	0	0	0	0	4	375	0
9. Other movements	2	4	1	18	14	38	2	1	0	278

(a)

Chest, 50 Hz

	0	1	2	3	4	5	6	7	8	9
0. Lying in the supine position	387	0	0	0	0	0	0	0	0	0
1. Standing	0	330	45	6	1	1	0	0	0	6
2. Sitting	0	73	305	5	1	1	1	0	0	3
3. Eating	0	12	6	316	10	31	0	0	0	19
4. Brushing teeth	0	1	3	10	348	1	0	0	0	24
5. Using the restroom	0	0	1	8	3	360	0	5	0	39
6. Walking	0	0	0	0	0	1	447	12	0	6
7. Ascending/descending the stairs	0	0	0	0	0	1	10	399	0	0
8. Running	0	0	0	0	0	0	0	5	374	0
9. Other movements	3	2	1	19	15	40	2	2	0	274

(b)

Chest, 25 Hz

	0	1	2	3	4	5	6	7	8	9
0. Lying in the supine position	387	0	0	0	0	0	0	0	0	0
1. Standing	0	329	45	5	2	1	0	0	0	7
2. Sitting	0	69	308	6	2	2	0	0	0	2
3. Eating	0	11	6	323	4	29	0	0	0	21
4. Brushing teeth	0	2	6	1	357	1	0	0	0	20
5. Using the restroom	0	0	1	10	3	355	1	2	0	44
6. Walking	0	0	0	0	0	1	450	10	0	5
7. Ascending/descending the stairs	0	0	0	0	0	1	6	403	0	0
8. Running	0	0	0	0	0	0	2	4	373	0
9. Other movements	2	3	2	19	15	42	2	2	0	271

(c)

Chest, 20 Hz

	0	1	2	3	4	5	6	7	8	9
0. Lying in the supine position	387	0	0	0	0	0	0	0	0	0
1. Standing	0	335	42	4	3	1	0	0	0	4
2. Sitting	0	76	303	7	0	2	0	0	0	1
3. Eating	0	16	7	321	2	28	0	0	0	20
4. Brushing teeth	0	1	3	4	362	1	0	0	0	16
5. Using the restroom	0	0	1	12	2	358	1	1	0	41
6. Walking	0	0	0	0	0	1	453	8	0	4
7. Ascending/descending the stairs	0	0	0	0	0	2	8	400	0	0
8. Running	0	0	0	0	0	0	2	4	373	0
9. Other movements	3	4	1	21	5	44	2	2	0	276

(d)

Chest, 10 Hz

	0	1	2	3	4	5	6	7	8	9
0. Lying in the supine position	387	0	0	0	0	0	0	0	0	0
1. Standing	0	335	41	6	2	1	0	0	0	4
2. Sitting	0	75	305	6	0	1	0	0	0	2
3. Eating	0	19	7	320	1	27	0	0	0	20
4. Brushing teeth	0	1	2	3	365	0	0	0	0	16
5. Using the restroom	0	0	1	11	2	360	2	1	0	39
6. Walking	0	0	0	0	0	1	456	4	0	5
7. Ascending/descending the stairs	0	0	0	0	0	1	2	406	1	0
8. Running	0	0	0	0	0	0	0	9	370	0
9. Other movements	2	2	2	21	5	43	0	1	0	282

(e)

Chest, 1 Hz

	0	1	2	3	4	5	6	7	8	9
0. Lying in the supine position	387	0	0	0	0	0	0	0	0	0
1. Standing	0	342	33	10	2	0	0	0	0	2
2. Sitting	0	75	290	0	24	0	0	0	0	0
3. Eating	0	19	3	267	44	35	0	0	0	26
4. Brushing teeth	0	4	14	27	315	1	0	0	0	26
5. Using the restroom	0	0	0	9	1	355	2	3	0	46
6. Walking	0	0	0	0	0	3	442	8	0	13
7. Ascending/descending the stairs	0	0	0	0	0	2	7	392	2	7
8. Running	0	1	0	0	0	1	1	1	375	0
9. Other movements	4	3	1	22	22	49	5	6	0	246

(f)
