# Supplementary material for: Effects of Sampling Frequency on Human Activity Recognition with Machine Learning Aiming at Clinical Applications
Source: Sensors (Basel). 2025 Jun 17;25(12):3780. doi: 10.3390/s25123780 (PMC12196717; doi:10.3390/s25123780)
Supplement: Supplementary file 1 [file sensors-25-03780-s001.zip › sensors-3635055-supplementary Table.pdf]

Supplementary materials

Table S1. Participant characteristics

Number of participants	30
Sex	13 males, 17 females
Mean age (years)	21.0 $\pm$ 0.87 (range: 19–23)
Handedness	29 right-handed, 1 left-handed
Health status	All healthy; no cardiovascular/respiratory conditions; non-pregnant
